# Reconstructing the initial global spread of a human influenza pandemic: A Bayesian spatial-temporal model for the global spread of H1N1pdm

**DOI:** 10.1371/currents.RRN1031

**Published:** 2009-09-02

**Authors:** Philippe Lemey, Marc Suchard, Andrew Rambaut

**Affiliations:** ^*^UCLA and ^†^Institute of Evolutionary Biology, University of Edinburgh, Edinburgh & The Fogarty International Center, NIH

## Abstract

Here, we present an analysis of the H1N1pdm genetic data sampled over the initial stages in the epidemic. To infer phylodynamic spread in time and space we employ a recently developed Bayesian statistical inference framework (Lemey et al., in press). We model spatial diffusion as a continuous-time Markov chain process along time-measured genealogies. In this analysis, we consider 40 locations for which sequence data were available on 06-Aug-2009. The sampling time interval of the 242 sequences spans from 30-Mar-2009 to 12-Jul-2009. The Bayesian inference typically results in a posterior distribution of phylogenetic trees, each having an estimate of the epidemic locations at the ancestral nodes in the tree. We summarize these trees using the most representative clustering pattern and annotate these clusters with the most probable location states. We can visualize this information as tree that grows over time, seeding locations each time an ancestral node is inferred to exist at a different location. A Bayes factor test provides statistical support for epidemiological linkage throughout the evolutionary history. We demonstrate how our full probabilistic approach efficiently tracks an epidemic based on viral genetic data as it unfolds across the globe.

## Introduction

First identified in April 2009, a novel Influenza A virus strain (H1N1pdm) of swine origin has taken on pandemic proportions in only a few months time. Since the initial H1N1pdm outbreak in Mexico, significant efforts have been undertaken to track the spread of the virus and to unravel its genetic make-up. The latter revealed multiple genetic ancestry of H1N1pdm, which could be tracked down to a triple-reassortant virus circulating in North American swine that combined gene segments form avian, human H3N2 and classical swine Influenza lineages [1] [2].

As part of the epidemiological surveillance, large amounts of H1N1pdm genetic sequences continue to accumulate. This represents an ideal opportunity to complement standard epidemiological monitoring with evolutionary inferences of viral gene flow in time and space. This contributes to a detailed characterization of H1N1pdm diffusion pathways from its origin to the current pandemic status. 

Here we employ Bayesian inference to sample reconstructed temporal and spatial histories from the posterior distribution given by the available genetic data. By not conditioning on a single reconstructed genealogy we are able to account for the uncertainty resulting from the limited genetic diversity in the H1N1pdm gene sequences providing estimates for metrics of interest that incorporate appropriate statistical errors. The approach also provides a conceptually intuitive framework in which to test competing models.

## Methods

To infer phylodynamic spread in time and space we employ a recently developed Bayesian statistical inference framework [3]. In this framework, we model spatial diffusion on time-measured genealogies as a continuous-time Markov chain (CTMC) process over discrete sampling locations. We fit this temporal-spatial process model simultaneously with well-established models of sequence evolution in a Bayesian genealogical approach using the software package BEAST [4] combined with the general phylogenetic likelihood evaluation library BEAGLE [5].  BEAGLE exploits many-core computing algorithms running on graphics processing units to achieve the marked speed-up necessary to analyze such a massive number of sequences across many locations.  By simultaneously integrating a CTMC model for discretized diffusion in BEAST, which is centered on time-scaled phylogenies, we infer spatial dynamics in real timescales.  To achieve statistical efficiency, we apply a Bayesian stochastic search variable selection (BSSVS) procedure that allows the CTMC rates to become zero with some positive, prior probability. Using such a procedure, the inference arrives at a parsimonious set of spatial diffusion rates that appropriately explain the spatial-temporal process. Further, comparing posterior to prior odds that individual rates are zero provides a formal Bayes factor test of the significance of spatial-temporal linkage between locations.

In this analysis, we consider sequence data available from the NCBI Influenza Database on 6th August 2009; our data sets consist of 242 sequences grouped according to 40 different locations and spanning a time interval from 30th March to 12th July. The data set is based on that compiled for Rambaut & Holmes (2009; [6]). The data consists of isolates for which at least the HA and NA genes were present but other genes were included when available, including 131 isolates with complete genomes sequenced. See Ref [6] for details of the sampling procedure. The sequence data used a Hasegawa-Kishino-Yano (HKY) substitution model with a discretized gamma distribution to model rate heterogeneity, and a relaxed molecular clock to account for variation in rates of evolution [7]. We ran Markov chain Monte Carlo (MCMC) analyses for two independent runs of 50 million generations, sampling every 10,000th generation; we investigated the stationarity of the distributions and the effective sample sizes of the MCMC chains using Tracer and combined the two runs after burnin to summarize the results. 

The Bayesian inference typically results in a posterior distribution of phylogenetic trees, and a tree traversal algorithm provides each ancestral node in those trees with a location drawn from the joint posterior distribution. Using TreeAnnotator, we summarize these trees using a representative clustering pattern (the "maximum clade credibility" tree, or MCC tree) and annotate these clusters with the most probable location states. Using GoogleEarth, we visualize this information as a tree that grows over time, seeding locations each time an ancestral node is inferred to exist at a different location.

## Results and Discussion

We summarize the Bayesian phylogeographic inference using the maximum clade credibility (MCC) tree in Figure 1; we annotate the most probable location for each node in the tree via color-labeling of the branches. Although the analysis considers 40 locations, including 24 US states, we only use color labels for 7 regions to capture the global patterns of H1N1pdm gene flu.

**Figure:**
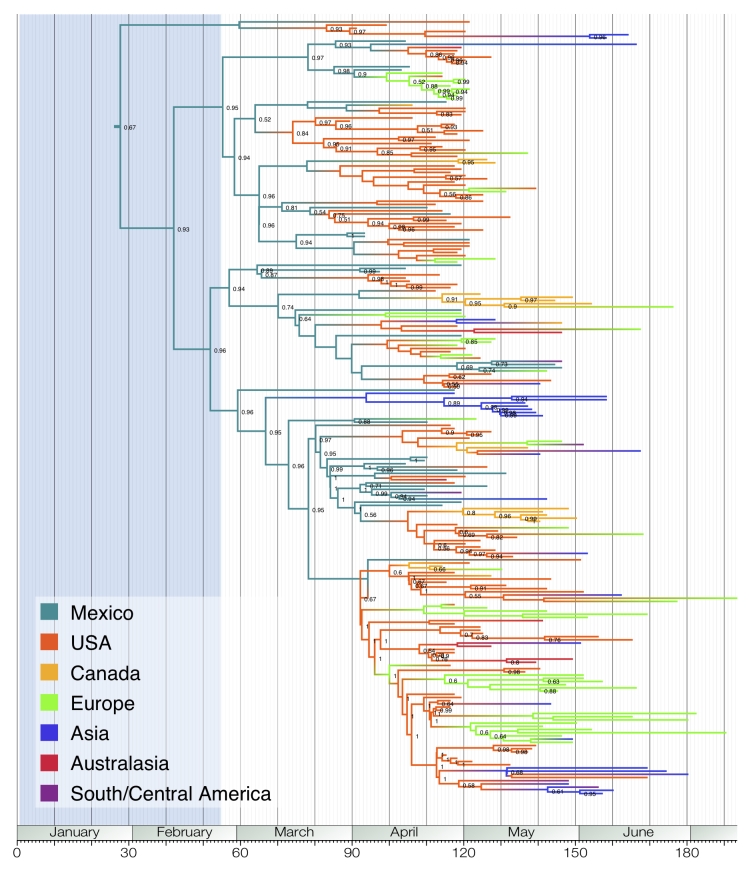
**Figure 1. **The maximum clade credibility (MCC) tree of H1N1pdm with CTMC spatial reconstruction. Lineages are coloured according to the highest posterior probability for location (these probabilities are shown when > 0.5). The blue band represents the 95% credible interval for the time of the most recent common ancestor.

The posterior distribution places notable probability on a Mexican origin for the most recent common ancestor (MRCA) and many subsequent nodes representing early ancestors in the tree. For the MRCA, this posterior probability is 0.67, compared to a prior probability of 0.025.  The change in prior to posterior odds is almost 80-fold.  While the early evolution most likely occurred in Mexico, the epidemic was independently seeding many US locations starting from around mid March, which results in a high density of US lineages in April. With a few exceptions of direct Mexico-Europe transitions, most European lineages appear to originate from the US.  The same holds true for Asian locations. The phylogeographic time-scale and other evolutionary parameters are very similar to the Bayesian coalescent analysis from Rambaut & Holmes (2009; [6]) 

**Table 1. **Marginal posterior estimates of model parameters.

**Table:** 

***parameter***	***mean (95% credible intervals)***
TMRCA (date)	27-Jan 09 (29-Dec 09,22-Feb 09)
evolutionary rate (subst./site/yr)	0.00496 (0.00410,0.00587)
growth rate (yr^-1^)	14.24 (9.64,18.52)
non-zero rates by BSSVS	43 (40,45)

To depict the global spread of the epidemic over time, Figure 2 reconstructs movements displayed sequentially over time.  An animated projection using GoogleEarth (Video 1) provides the same inferred spatial-temporal process.

**Figure:**
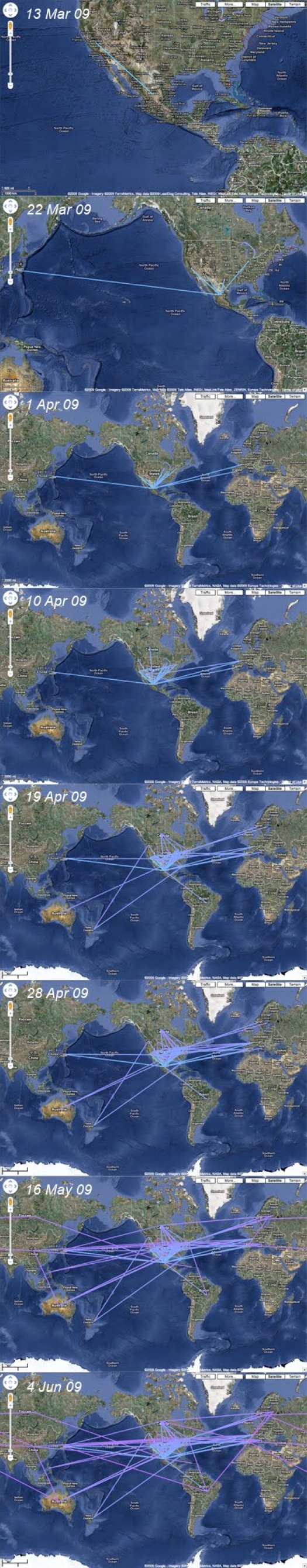
**Figure 2. **Temporal H1N1pdm dynamics represented by MCC projections over time. Branches which start and finish at different locations are shown at their temporal midpoint. To show the sequence of migrations, branches earlier in the epidemic are in blue, later in purple.

**Video 1:**
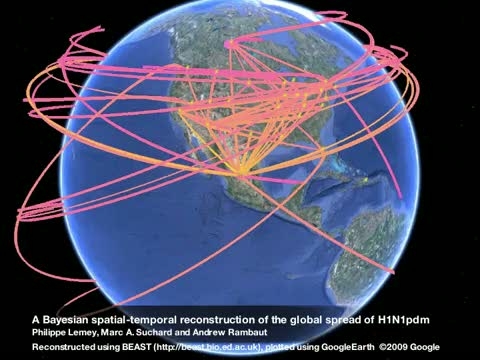
Temporal H1N1pdm dynamics visualized using Google Earth. Branches are shown traversing arcs between reconstructed locations over the time they span.

 Although the MCC projections in Figure 2 and Video 1 provide a glimpse into H1N1pdm spatial diffusion over time, they depict a scenario derived from a single tree in the posterior distribution. To establish epidemiological linkage while taking into account phylogenetic uncertainty, we perform BSSVS using a prior that prefers a minimal set of location exchange rates to explain the diffusion patterns (a truncated Poisson prior with mean = log2 and offset = 39; see [3]). This analysis results in a posterior mean of 43 (40-45) non-zero rates (Table 1). We summarize rates yielding a Bayes factor > 5 in Figure 3 & 4, which reveal epidemiological linkage globally and within North America respectively. The strongest link is observed between Mexico and the state of Texas (BF=74). Mexico is involved in only two additional links with Bayes factor > 5. The earliest dispersal event between Mexico and California, as suggested by the MCC tree in Figure 2, does not yield convincing support, most probably because this occurred deeper in the tree. Whereas locations like Australia and New Zealand are directly linked to North America, China can only be indirectly connected to North America (via either Russia, Europe or South America). Europe has multiple links with North America and Canada, but Scandinavian countries also establish epidemiological linkage with South America, China and Russia. Noteworthy is the multitude of epidemiological linkage for Brazil, including a strong link with Japan and other connections suggested with New York, Finland, Russia, Spain, China and Canada. The former can be explained by the fact that Brazil has the largest community of Japanese and their descendants outside Japan, while the latter may be the result of sink dynamics in the form of holiday travel. Within North America, both short and long distance connections make up a complex network of epidemiological linkage.

**Figure:**
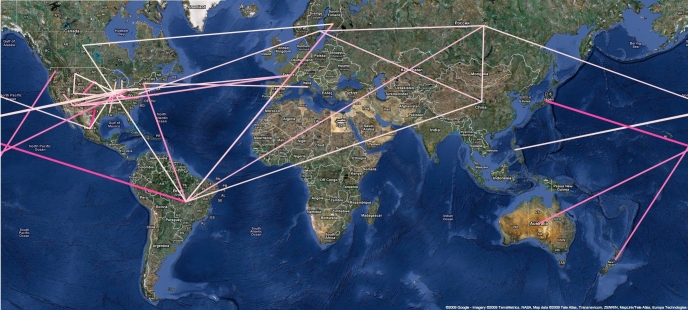
**Figure 3. **Significant global rates of movement identified by BSSVS. The color of the connections represent the relative strength by which the rates among these two locations are supported: white = weak, magenta = strong.

**Figure:**
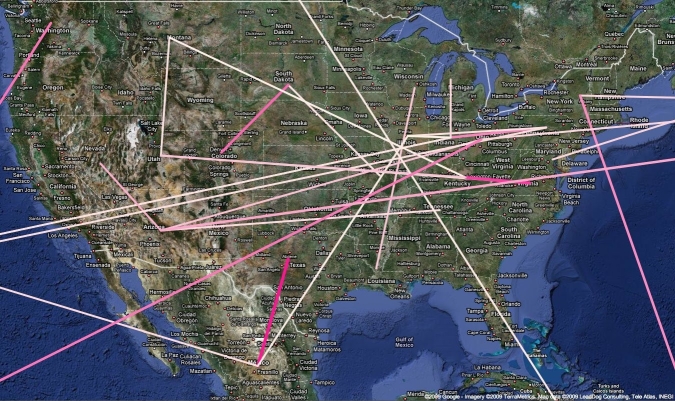
**Figure 4.** A more detailed view of significant linkage in North America.

Genetic sequences provide a unique source of information about the epidemiological connections between viruses sampled through time and across space. Viral genetics can illuminate the obscure origins of an epidemic, for example, before an infectious agent was identified and track its global trajectories once it has grown too large for individual cases to be examined. Fortunately, recent many-core computational algorithms [5] make possible these forms of joint analyses in a Bayesian framework.  Along these lines, the formal spatial-temporal process model we develop uniquely ties together sequence evolution and geographic history in a statistically coherent approach. The general framework also emits an easy incorporation of more detailed geographic information, such as air-traffic data, as a prior distribution on the rates of location exchange and comparison of competing geographic models using standard Bayesian model selection techniques.

**Acknowledgments**

 Thanks to Gytis Dudas for help with collating the sequence data. 

**Funding information**

PL was funded by a postdoctoral fellowship from the Flemish Science Foundation (FWO) and FWO grant G.0513.06. MAS was funded by the John Simon Guggenheim Memorial Foundation and NIH R01 grant GM086887. AR was funded by The Royal Society of London and the Interdisciplinary Centre for Human and Avian Influenza Research (ICHAIR).  Further thanks are owed to the Fogarty International Center, NIH, for support.

**Competing interests**

 The authors have declared that no competing interests exist. 
